# Use of ultraviolet-light irradiated multiple myeloma cells as immunogens to generate tumor-specific cytolytic T lymphocytes

**DOI:** 10.1186/1476-8518-6-2

**Published:** 2008-04-28

**Authors:** Charles A Gullo, William YK Hwang, Chye K Poh, Melvin Au, Geraline Cow, Gerrard Teoh

**Affiliations:** 1 Cancer Immunology Laboratory, Department of Clinical Research, Singapore General Hospital, Outram Road, 169608 Singapore; 2 Multiple Myeloma Research Laboratory, SingHealth Research Facilities, 7 Hospital Drive, Block A #02-01, 169611 Singapore; 3 Department of Hematology, Singapore General Hospital (SGH), Outram Road, 169608 Singapore

## Abstract

**Background:**

As the eradication of tumor cells *in vivo *is most efficiently performed by cytolytic T lymphocytes (CTL), various methods for priming tumor-reactive lymphocytes have been developed. In this study, a method of priming CTLs with ultraviolet (UV)-irradiated tumor cells, which results in termination of tumor cell proliferation, apoptosis, as well as upregulation of heat shock proteins (HSP) expression is described.

**Methods:**

Peripheral blood mononuclear cells (PBMC) were primed weekly with UV-irradiated or mitomycin-treated RPMI 8226 multiple myeloma cells. Following three rounds of stimulation over 21 days, the lymphocytes from the mixed culture conditions were analyzed for anti-MM cell reactivity.

**Results:**

By day 10 of cultures, PBMCs primed using UV-irradiated tumor cells demonstrated a higher percentage of activated CD8+/CD4- T lymphocytes than non-primed PBMCs or PBMCs primed using mitomycin-treated MM cells. Cytotoxicity assays revealed that primed PBMCs were markedly more effective (p < 0.01) than non-primed PBMCs in killing RPMI 8226 MM cells. Surface expression of glucose regulated protein 94 (Grp94/Gp96) and Grp78 were both found to be induced in UV-treated MM cells.

**Conclusion:**

Since, HSP-associated peptides are known to mediate tumor rejection; these data suggest that immune-mediated eradication of MM cells could be elicited via a UV-induced HSP process. The finding that the addition of 17-allylamide-17-demethoxygeldanamycin (17AAG, an inhibitor of HSP 90-peptide interactions) resulted in decreased CTL-induced cytotoxicity supported this hypothesis. Our study, therefore, provides the framework for the development of anti-tumor CTL cellular vaccines for treating MM using UV-irradiated tumor cells as immunogens.

## Background

Multiple myeloma (MM), a malignancy of terminally differentiated plasma cells, is the most common hematologic cancer in the United States (US) [1]. Currently, MM is incurable and there is an urgent need for the development of novel and curative forms of therapy, including immunotherapy. A major limitation on the treatment of all cancers is the evasion strategy developed by tumor cells to bypass immune surveillance. Thus far, specific anti-cellular therapy against MM has been difficult to develop because of the down regulation of tumor cell surface antigens (Ag) and the attenuation of host immunity by MM cells. The majority of MM cellular immunotherapies have focused on deriving anti idiotypic (Id) patient-specific CTL [2-4]. Other strategies include the use of MM-specific RNA transduced dendritic cells and MM apoptotic bodies pulsed dendritic cells [5,6]. Unfortunately, although some of these studies were promising none of them have achieved high response rates and therefore have not been actively pursued.

As eradication of tumor cells *in vivo *is most efficiently performed by CD8^+ ^and/or CD4^-^CD8^- ^natural killer (NK) cytolytic T lymphocytes (CTL) [7,8], various methods for increasing recognition by lymphocytes have been developed. Since the success of such therapies depends significantly on the efficiency of Ag presentation, tumor cells have been genetically modified to present antigens directly to tumor reactive T cells [9]. Other potentially safer methods include the use of cytokines and/or adjuvants to increase the antigenicity of the tumor itself or to enhance the immune recognition of the tumor *in vivo *[2]. Reinfusion of tumor-specific CTLs as therapy against cancer (i.e. adoptive immunotherapy) has been used in the treatment of a number of cancers [10] proving particularly effective in some patients with melanoma [11]. Several groups have also recently characterized a novel class of cells that have potent innate anti-tumor properties termed Cytokine Induced Killer (CIKs) [12] or Interferon-γ producing Killer Dendritic Cells (IKDCs) [13]. Those as well as newly developed anti-tumor cytolytic cells with NK cell markers [14] demonstrate renewed interest in finding non antigen-specific anti-tumor effector cells for cellular adoptive therapy.

Unfortunately, searches for novel tumor-associated (TAAs) for many cancers have been extremely slow and those that do exist have rarely translated into clinically effective therapies [15]. Thus finding a source of tumor-specific antigens with adjuvant properties that can be used in an adoptive-immunotherapy setting to elicit strong T cell responses is greatly desired. In a landmark paper by Srivastava *et al*., tumor antigens were isolated from chemically induced sarcomas and found to be capable of mediating tumor rejection [16]. One of these tumor antigens was identified as tumor rejection Ag-1 (TRA-1, gp96, Grp94), a heat shock protein (HSP)-90 family member [17], which is capable of mediating tumor rejection of various cancers. Heat shock proteins are induced when cells are exposed to stress (e.g. DNA damage) [18], and have now been intimately linked to antigen presentation pathways [19,20]. In fact, HSPs Grp94, HSP-90, and Grp78 are known to carry peptides that are stimulatory to CD8^+ ^CTL via a process known as cross-presentation [21] and are being currently used for immunotherapy [22-24]. Expression of HSP can be induced using ultraviolet (UV) light irradiation [25], which damages DNA and triggers the stress response in the cell [26]. Hence, we embarked on this study to determine if UV-irradiation could be used as a means to concomitantly increase HSP expression and tumor cell antigenicity of MM cells, resulting in the production of effective anti-tumor CTLs. We demonstrate that UV-irradiation indeed induces total RNA, protein, as well as surface expression of Grp94 and Grp78, two potent immunogenic HSPs. Furthermore, UV-irradiated MM cells were shown to be effective in priming CD8^+ ^CTL and resulted in efficient induction of anti-MM CTL *in-vitro*. Finally, recognition of the UV-irradiated tumor cells by the CTL was partially inhibited by the ansymcin antibiotic 17-AAG, an HSP-protein inhibitor [9,16,18].

## Methods

### Cells

The human RPMI 8226 MM (CCL-155) cell line was purchased from American Type Culture Collection (ATCC, Rockville, MD) and cultured in complete media consisting of 90% RPMI 1640 with L-glutamine media, 10% fetal bovine serum (FBS), 25 IU/ml penicillin, 25 μg/ml streptomycin, and additional 5 mM L-glutamine. Normal human peripheral blood (PB) was obtained from volunteer donors with informed consent and under institutional review board (IRB) approval (Singapore General Hospital, IRB approval #103/2003). Peripheral blood mononuclear cells (PBMCs) were isolated using Ficoll-Hypaque (Amersham Pharmacia, Uppsala, Sweden) density gradient sedimentation and cultured in complete media. Cell cultures were maintained at 37°C with 5% CO_2 _in a humidified atmosphere. Cells were enumerated using standard trypan blue (Gibco BRL, Life Technologies, Carlsbad, CA) exclusion assays.

### UV-irradiation of RPMI 8226 MM cell line

UV-irradiation was performed using the Stratagene Stratalinker UV Crosslinker (Stratagene, La Jolla, CA). For various experiments, UV-irradiation (6 mJ/cm^2 ^to 240 mJ/cm^2^) was performed on open 10 cm diameter tissue culture dishes containing RPMI 8226 MM cells (1.5 × 10^6 ^in 3.0 mL of complete media).

### Cell proliferation assays

Cell proliferation was assessed using DNA synthesis assay. Here, standard tritiated thymidine (^3^H-TdR, Perkin Elmer Life Sciences, Boston, MA) incorporation assays were performed. Cells (5,000 cells/mL) were first incubated with 0.25 μCi/well overnight and then treated for 3 hrs in 96-well tissue culture plates (200 μL/well) with various doses of UV-irradiation or left untreated. Next, cells were harvested onto fiberglass filters using a cell harvester (Tomtec Mach III Auto, Tomtec, Hamden, CT) and counted on a beta plate reader (Wallac 1450 MicroBeta TriLux, Turku, Finland). Each proliferation result is an average of three independent experiments. Readings are expressed as counts per minute (CPM) and derived from triplicate values for each condition. No proliferation was observed in long-term cultures for cells exposed to 120 mj/cm2 or above UV-irradiation.

### Annexin V/propidium iodide (PI) staining

In order to assay for the presence of apoptosis, RPMI 8226 MM cells (1 × 10^5^/sample) were dually stained using fluorescein isothiocynate (FITC) labeled annexin V (annexin V-FITC) and PI (BD Pharmingen, San Diego, CA) according to the protocol provided by the manufacturer. Briefly, cells were washed and suspended in 100 μL of binding buffer, then stained using 5 μL of annexin V-FITC and 2 μL of PI. Analysis was performed on the Cytomics FC500 flow cytometric analyzer (Beckman Coulter, Miami, FL). In this assay, annexin V^+^/PI^- ^cells represent MM cells in early stages of apoptosis, whereas dually positive annexin V^+^/PI^+ ^cells are MM cells that have undergone apoptosis. Data from each flow histogram quadrant (Annexin-V FITC detected in the first channel and PI in the second channel) is then tabulated and represented as % apoptosis in chart form.

### Priming of PBMCs under 'mixed-culture' conditions

Priming of PBMCs was performed by co-culture of PBMCs (5 × 10^5^/well) and UV-irradiated (120 mJ/cm^2^, 5 × 10^6^/well) or mitomycin (100 μg/ml, 5 × 10^6^/well) treated RPMI 8226 MM cells in 6-well tissue culture plates. Thus the responder cell to stimulator cell ratio was 1:10. Cell co-cultures were maintained in complete media with recombinant human interleukin-2 (IL-2, R & D Systems, Minneapolis, MN, 0.5 ng/mL). Weekly priming (Days 0, 7, 14, 21 and 28) was performed by adding UV-irradiated or mitomycin-treated RPMI 8226 MM cells (5 × 10^6^/well) into the respective existing co-culture. Half-media exchanges containing IL-2 were performed every 3 days to 5 days. Cells were collected and washed weekly before each 'mixed-culture' priming was performed.

### Indirect fluorescence flow cytometric analysis

T cell subset immunophenotyping and HSP expression were performed using indirect fluorescence flow cytometric analysis (Cytomics FC500 flow cytometric analyzer, Beckman Coulter). The following mouse anti-human monoclonal antibodies (mAbs) were used for effector cell immunophenotyping -anti-CD8, anti-CD45, anti-CD56, anti-granzyme A, and anti-perforin (Beckman Coulter). Antibodies were directly conjugated with fluorophores (FITC and phycoerythrin (PE)). Lymphocytes were identified as CD45^bright ^plus low side-scatter cells. For assessment of heat shock protein expression, goat anti-Grp78 (C-20, sc-1051) and goat anti-Grp94 (C-19, sc-1794) (both from Santa Cruz Biotechnology, Santa Cruz, CA) were used. The secondary antibody used (where appropriate) was donkey anti-goat FITC mAb (Santa Cruz). Briefly, primed PBMCs or UV-irradiated RPMI 8226 MM cells (0.5 × 10^6 ^to 1.0 × 10^6^/sample) were harvested, washed three times in phosphate buffered saline (PBS), blocked with 10% human AB serum in PBS, and stained using the appropriate mAb at 4°C for 30 mins. Next, cells were washed three times in PBS prior to analysis. Statistical analysis was performed using the Chi-squared test (Microsoft Excel, Microsoft Office 97, Microsoft Corp., Redmond, WA).

### CTL assay

A tritiated thymidine (^3^H-TdR)-based cytotoxicity assay (JAM assay) was used to evaluate the killing of viable RPMI 8226 MM cells by primed and non-primed PBMCs [27]. In this assay, ^3^H-TdR is first incorporated into DNA during labeling. During analysis, degraded DNA is washed through a fiberglass filter, leaving behind the intact, high molecular weight DNA. Briefly, RPMI 8226 MM cells (target cells, T) were first labeled with ^3^H-TdR (10 μCi/ml, PerkinElmer Life Sciences, Waltham, MA USA) for 12 hrs in 96-well tissue culture plates (2 × 10^4 ^cells/well). Primed or non-primed PBMCs (effector, E) were co-cultured with target cells at various E:T (0:1, 25:1, 50:1, 75:1 or 100:1) ratios for various times. Next, cells were harvested onto fiberglass filters using a cell harvester (Tomtec Inc. Hamden, CT USA) and counted on a beta plate reader (PerkinElmer,). The percentage of specific killing relative to medium-stimulated controls was calculated as [(Spontaneous – Experimental)/Spontaneous] × 100], where the radioactivity (cpm) in T cells exposed to control medium was defined as S and that of treated cells was defined as E [28].

### Western immunoblotting

Whole cell extracts (WCE) were obtained from non-UV-irradiated and UV-irradiated RPMI 8226 MM cells (3.0 × 10^6 ^cells/sample) using EBC1 lysis buffer, which contains 50 mM Tris pH 8.0, 150 mM NaCl, 0.1% NP-40, 0.5 μg/mL phenylmethylsulfonyl fluoride (PMSF), 50 mM NaF, 1 mM NaVO_4_, and a Complete^® ^protease inhibitor tablet (Roche Diagnostics GmbH, Mannheim, Germany) in every 50 mL. Proteins were quantified using Bradford's method (Bio-Rad, Hercules, CA), and resolved (20 μg/lane) in a 12% sodium dodecyl sulphate-polyacrylamide gel electrophoresis (SDS-PAGE) gel. Next, proteins were transferred to a polyvinylidene fluoride (PVDF) membrane (Schleicher & Schuell, Keene, NH), and blocked for 2 hrs using Tris-buffered saline (TBS) – 20 mM Tris pH 7.6, 150 mM NaCl – containing 1.0% Tween-20 (Sigma-Aldrich, St Louis, MO). Membranes were probed using goat anti-Grp78 (C-20, sc-1051) or goat anti-Grp94 (C-19, sc-1794) mAbs 1:200 dilution (Santa Cruz Biotechnology, Santa Cruz, CA) for 1 hr, then washed thrice with TBS containing 0.2% Tween-20 (TBST), and then reprobed using horseradish peroxidase (hrp)-conjugated donkey anti-goat IgG mAb 1:15,000 dilution (Santa Cruz Biotechnologies) for 2 hrs. Next, membranes were washed 6 times with TBST, and chemiluminescene detection was preformed using ChemiGlow^® ^reagents and filmless imaging on the FluoChem Imager™ (both from Alpha Innotech, San Leandro, CA). Spot densitometry was performed using AlphaEaseFC™ software (Alpha Innotech). In some cases, to determine relative expression, the normalized values for each condition were derived by taking the integrated density values from the protein in question and dividing that by the values for Actin in that condition.

### Quantitative Real Time Reverse Transcription PCR (Q-PCR)

RNA was extracted from the RPMI 8226 MM cell line using the RNeasy RNA Extraction Kit (Qiagen Gmb, Hilden, Germany) according to the manufacturer's recommendations, and quantified using the GeneQuant Pro Kit (Amersham Biosciences, Piscataway, NJ). Quantitative real time revere transcription-PCR (250 ng of RNA/sample) was performed using the Roche LightCycler^® ^system (Roche Diagnostics, 20 μL/reaction) for grp78 using the following primers (25 nM each primer): 5'-CCCTCACTATGAATGGGT-3' (forward), and 5'-GTGATCTCGGCTCACT-3' (reverse); as well as grp94 using the following primers: 5'-CTGAAAAAGGGCTATGAAGT-3' (forward), and 5'-CCTTGCCGGTTTGGTA-3' (reverse). Amplification of β-actin was used as an internal control; 5'-ATCTGGCACCACACCTTCTAGCAA TGAGCTGCG-3' (forward); 5'-CGTCATACTCCTGCTTGCTGA TCCACATCTGC-3' (reverse). Reaction conditions were 94°C 1 s denaturation, 64°C 1 s annealing, and 72°C 15 s extension; for a total of 40 cycles. Human grp94 and grp78 as well as, β-actin PCR efficiencies were calculated as 10^-1/slope^, where the slope of the line was determined from the relationship between PCR crossing point and the logarithm of concentration. The adjusted efficiencies for each gene was determined by 5 individual tissue samples diluted over five 10-fold concentrations. The relative ratio for each HSP was calculated using unknown and calibrator RNA using the following formula: (where c = calibrator and U = unknown and CP = crossing point)

$$\frac{{\text{Efficiency (h-HSP)}}^{\text{CP(hHSP.C-hHSP.U)}}}{{\text{Efficiency (β-actin)}}^{\text{CP(b-actin.C-b-actin.U)}}}$$

Data was analyzed using the Relative Quantitation software (Roche Diagnostics) and is expressed as relative expression.

## Results and Discussion

### Effects of UV-irradiation on the proliferation and apoptosis of RPMI 8226 cell line

**Figure 1:**
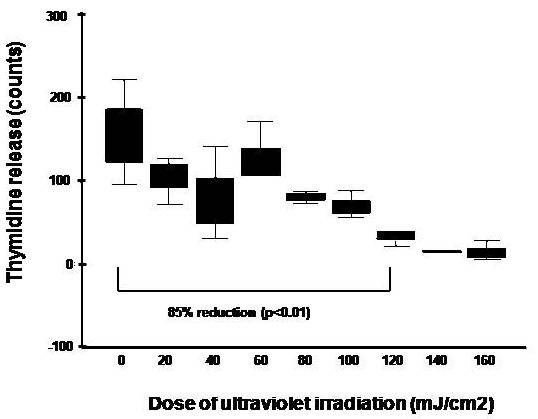
**Effect of UV-irradiation on short-term proliferation of RPMI 8226 MM cell line**. The RPMI 8226 MM cell line was irradiated using UV light (0, 20, 40, 60, 80, 100, 120, 140 or 160 mJ/cm^2^) and assessed for DNA synthesis using standard ^3^H-TdR (0.25 μCi/well) incorporation assays 3 hr after UV-irradiation. Three triplicate experiments were performed and data is shown as box plots. Statistical differences were determined using the Mann-Whitney test.

Ultra violet irradiation can be an effective sterile and non-pharmacological way to induce apoptosis of tumor cells. Although UV-irradiation of tumor cells results in genotoxic damage and death *in vitro, in vivo *irradiation can result in impairment of tumor rejection in mice and possibly in humans due to impairment of the tumor-specific immunocytes [29]. Thus, determining the dose of irradiation for treating tumors ex *vivo *is important and preferred over localized or total body irradiation as a means of reducing tumor burden and developing effective anti-tumor immune responses. In order to determine the minimal dose of UV-irradiation that will inhibit tumor cell growth, we first compared the effect of different doses (0, 20, 40, 60, 80, 100, 120, 140 or 160 mJ/cm^2^) of UV-irradiation on short-term (3 hr) proliferation in the RPMI 8226 MM cell line. As can be seen in Figure 1, there is a statistically significant (p < 0.01) dose dependent decrease in tumor cell growth at all dose levels. These data suggest that the minimal UV-irradiation dose required to inhibit RPMI 8226 MM cell line growth was 120 mJ/cm^2^. Thus, at 120 mJ/cm^2 ^dose, the proliferation of RPMI cells is inhibited by approximately 85% in 3 hours and would therefore unlikely outgrow the cultures when mixed with PBMC.

Since, lymphocyte priming requires long-term cell co-culture with UV-irradiated MM cells, we next compared the effect of different doses (6, 12, 24, 60, 120 or 240 mJ/cm^2^) of UV-irradiation on MM cell proliferation for up to 8 weeks. Importantly, a low UV dose that prevents overgrowth of cell cultures by the surviving MM cells but that still maintain tumor antigen presentation was selected, as this maneuver would facilitate antigen recognition by T lymphocytes [30]. The minimal UV- irradiation dose that will inhibit long-term growth of RPMI 8226 MM cell line is also 120 mJ/cm^2 ^(data not shown). Hence, this dose of UV-irradiation was used in subsequent experiments.

**Figure 2:**
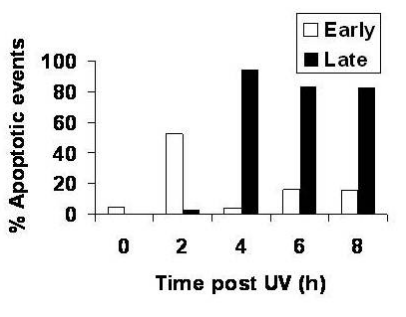
**Effect of UV-irradiation on apoptosis of RPMI 8226 MM cell lines**. In order to determine the time of onset of apoptosis of the RPMI 8226 MM cell line following UV-irradiation, tumor cells were first irradiated with 120 mJ/cm2 of UV light and serially (0, 2, 4, 6 and 8 hr post UV-irradiation) analyzed for the onset of early apoptosis (annexin V+PI-) or established apoptosis (annexin V+PI+) using annexin V-FITC/PI dual staining and fluorescence flow cytometric analysis. All experiments were performed triplicate and expressed as mean values.

For the development of an effective antigen-presentation dependent vaccine, a treatment that results in apoptosis and not necrosis of the tumor itself can often be highly desired [31-34]. Having determined the optimal UV dose (i.e. 120 mJ/cm^2^) that would result in growth arrest of the RPMI 8226 MM cell line, we next determined the time to onset of apoptosis of tumor cells following UV-irradiation. As can be seen in Figure 2, 52.4% of RPMI 8226 MM cells exposed to 120 mJ/cm^2 ^of UV-irradiation demonstrated cells present in the early phases of apoptosis (annexin V^+^PI^-^) with fewer cells present at the later stage of apoptosis by two hours post UV-irradiation. However, by four hours post UV-irradiation, 94.5% of tumor cells had undergone established apoptosis (annexin V^+^PI^+^) and this was accompanied with by a concomitant decrease in cells found in early phases of apoptosis. There was also little PI-only staining cells by four hours (data not shown), suggesting that little necrosis has occurred at this point. These data confirm that UV-irradiation is an effective way to inhibit proliferation and induce apoptosis of the RPMI 8226 MM cell line, and that apoptosis is best achieved with a short dose time of UV-irradiation at an optimized dose of 120 mj/cm^2^.

### Expansion of CD8^+ ^CTLs by UV-irradiated RPMI 8226 MM under 'mixed-culture' priming conditions

**Figure 3:**
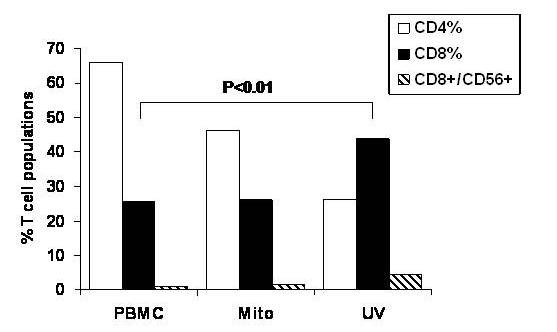
**Expansion of CD8^+ ^CTLs by UV-irradiated RPMI 8226 MM cell whole-cell immunogens in 'mixed culture' conditions**. Normal donor PBMCs were primed using UV-irradiated (120 mJ/cm^2^) or mitomycin-treated (100 μg/mL) RPMI 8226 MM cells as whole cell immunogens for 10 days and analyzed for CD4, CD8, CD56, granzyme and perforin expression using indirect immunofluorescence flow cytometric analysis. Unprimed and concurrently cultured PBMCs were used as negative controls. Dually positive granzyme^+^perforin^+ ^CTLs were identified gated and specifically analyzed for CD4^+ ^(open columns), CD8^+ ^(black columns) or CD8^+^/CD56^+ ^(striped columns) expression.

In order to determine whether optimally UV-irradiated RPMI 8226 MM cells could be used as whole cell immunogens for PBMC priming, we studied the lymphocyte subsets that were generated after 10 days of PBMC priming. Priming was performed with four hour UV-treated and thus apoptosis-induced MM cells. As can be seen in Figure 3, priming of PBMCs using UV-irradiated RPMI 8226 MM cells led to a significant (p < 0.01) expansion of CD8^+ ^CTLs (43.8%) when compared to unprimed PBMCs (25.7%) or PBMCs primed using mitomycin-treated RPMI 8226 MM cells (26.2%); together with a reciprocal decrease in CD4^+ ^CTLs. In addition, there was also a small increase (4.43%) in the CD8^+^/CD56^+ ^NK cell population as compared to unprimed PBMCs (1.16%) or PBMCs primed using mitomycin-treated RPMI 8226 MM cells (1.65%). In contrast, mitomycin-treated tumor cell immunogens were not associated with any significant expansion of CD4^+^, CD8^+ ^or CD8^+^/CD56^+ ^CTLs, when compared to unprimed PBMCs. This increase in CD8^+ ^CTL was sustained during the 21-day co-culture and survival was ensured by the addition of exogenous recombinant human IL-2 (data not shown) [35].

### Efficient CTL redirected lysis of fresh RPMI cells following 'mixed-culture' T cell priming conditions

**Figure 4:**
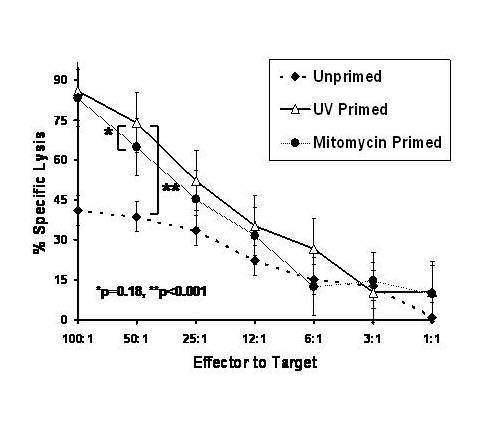
**Redirected CTL assay against RPMI following mixed culture conditions with UV-primed RPMI cells**. Normal donor PBMCs were primed using UV-irradiated (120 mJ/cm^2^) or mitomycin-treated (100 μg/mL) RPMI 8226 MM cells as whole cell immunogens for 28 days in 'mixed-culture conditions'. Non-primed, mito-primed, or UV-primed and PBMCs (effector cells) were then co-cultured with viable ^3^H-TdR-labeled RPMI 8226 MM cells (target cells) in a re-directed CTL assay. A standard ^3^H-TdR cytotoxicity assays was performed at E:T ratios of 1:1, 3:1, 6:1, 12:1 or 25:1, 50:1 and 100:1.

In order to determine whether CD8^+ ^CTLs were effective in eradicating MM cells, we next performed cytotoxic assays using viable RPMI 8226 MM cells as target cells. As can be seen in Figure 4, PBMCs primed using UV-irradiated or mitomycin (mito)-treated RPMI 8226 MM cells in the 'mixed-culture' regimen were effective in eradicating fresh RPMI 8226 MM. Maximal lysis of RPMI 8226 MM cells was most marked with PBMC primed with UV-RPMI (84.65 to 88.19%, median 85.26%) and less marked when primed with mito-RPMI (82.59 to 83.80%, median 83.33%), although the difference was not statistically significant (p = 0.18). UV-RPMI or mito-RPMI primed PBMC demonstrated maximal lysis, which was significantly (p < 0.001) more than the unprimed PBMC (38.02% to 43.19%, median 42.38). These results indicate that the priming of allogeneic PBMC with UV irradiated MM cells results in the marked anti-tumor cytolytic activity, which is at least as good as the priming with mitomycin, inactivated MM cells. When the primed cells were co-cultured with autologous PBMC cells, little RPMI directed cytotoxic cell lysis was seen (data not shown).

### Up regulation of heat shock protein expression on RPMI-8226 cells following UV-irradiation

**Figure 5:**
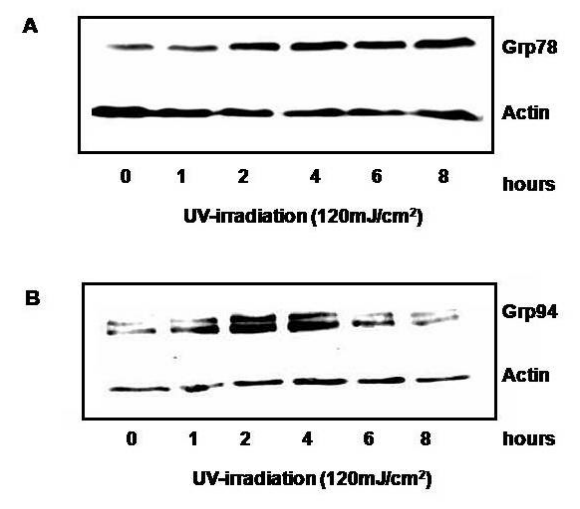
**Effect of UV-irradiation on total Grp78 and 94 protein expression**. Whole cell lysates of RPMI-8226 cells at 1, 2, 4, 6, and 8 hours after an UV-irradiation dose of 120 mJ/cm2, were run on a 12% polyacrylamide gel. Western immunoblotting was performed with anti-Grp78 (A) and anti-Grp94 (B) antibodies and data is representative to two independent experiments.

Since the upregulation of heat shock protein (HSP)/molecular chaperones, especially Grp94 and 78, can promote the antigenicity of tumor cells and since ultraviolet irradiation can potentially induce the expression of HSPs; we went on to investigate if the same doses of UV-irradiation resulted in an increase in heat shock proteins on the RPMI tumor cells. Upon western blot analysis, expression of the cytosolic HSP-70 protein, Grp78, was increased after UV-irradiation at a dose of 120 mJ/cm^2 ^by two hours (Figure 5A). Using polyclonal goat antibodies reactive against the endoplasmic reticulum HSP-90 protein, Grp94, there was a marked increase in the two faster migrating bands that reacted with the anti-Grp antibody (Figure 5B). Although there was no change in the slower migrating bands recognized by our anti-Grp94 antibodies, there was an increase in the total amount of Grp94 seen by Western immunoblotting. This increase was transient and reached a maximum by four hours. Due to the observed slight transient increase in the Actin levels, integrated density values were determined and the Grp94 density was calculated after normalization with Actin at each condition. The values still showed a transient increase in Grp94 (values were 0.851, 2.09, 3.25, 2.38, 1.09, and 0.777 for each time point).

**Figure 6:**
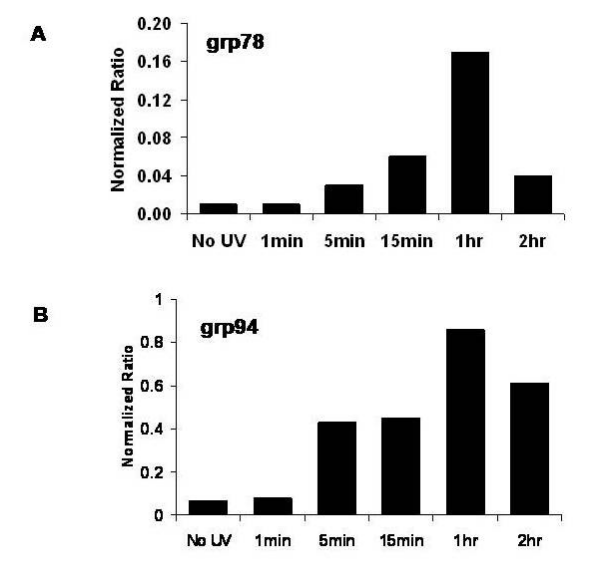
**Effect of UV-irradiation on mRNA expression of Grp94 and Grp78 in RPMI 8226 cells**. RPMI 8226 cells were subjected to UV-irradiation at 120 mJ/cm2 and RNA was extracted 1, 15, 60 and 120 minutes. Real-time PCR using actin and grp78 (A) or grp94 (B) primers was performed and data is expressed as calibrator corrected and actin normalized ratios.

In order to determine if UV-irradiation was accompanied by an increase in heat shock protein mRNA expression, relative expression of grp94 and grp78 mRNA before and after UV-irradiation by quantitative real-time polymerase chain reaction (RT-PCR) was performed. As seen in Figure 6A, grp78 mRNA expression rapidly increased 17-fold by 1 hour after 120 mJ/cm^2 ^UV-irradiation, falling off by 2 hours. Similarly, grp94 mRNA expression increased 12.3-fold 1 hour after UV-irradiation, subsequently decreasing by 2 hours post UV treatment (Figure 6B). Thus, UV-irradiation induces early heat shock protein message expression followed later by cell protein expression.

**Figure 7:**
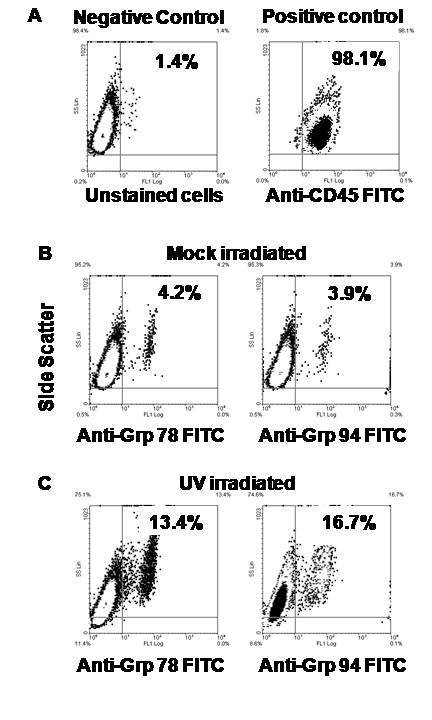
**Cell surface expression of MHC molecules afterUV-irradiation**. RPMI-8226 cells were subjected to 120 mJ/cm2 of UV-irradiation. Cells were then stained with anti-Grp94 and anti-Grp78 antibodies. The figures show a plot of side scatter on the vertical axis versus FL-1 on the horizontal axis of the dot plot. Cells were stained with anti-CD45 (as control (A)), anti-Grp94 (B), and anti-Grp78 (C) following four hours of unstimulated or UV-irradiated conditions.

Heat shock proteins are expressed in the cell membrane during cell stress or in abnormal cells such as tumor cells [36,37] but not in non-neoplastic cells. It is likely that surface expressed heat shock proteins contain immunogenic peptides that are taken up by receptors on APCs and can be cross-presented onto cytolytic T lymphocytes [19]. In order to determine in RPMI cells express Grp78 or Grp94 on their cell surface following UV exposure, the cells were stained with antibodies against these HSPs and analyzed by flow cytometry. Although the cells contained a small amount of both Grp78 (4.25%) and Grp94 (3.9%), both were dramatically increased upon UV stimulation after four hours (Figure 7). Therefore, UV-exposure of RPMI after four hours induces early apoptosis, arrests proliferation, results in efficient priming of CTL and is accompanied by increased protein and messenger RNA expression of heat shock proteins Grp78 and 94.

### HSP-peptide inhibitors block UV-irradiated RPMI recognition by cytolytic T cells

**Figure 8:**
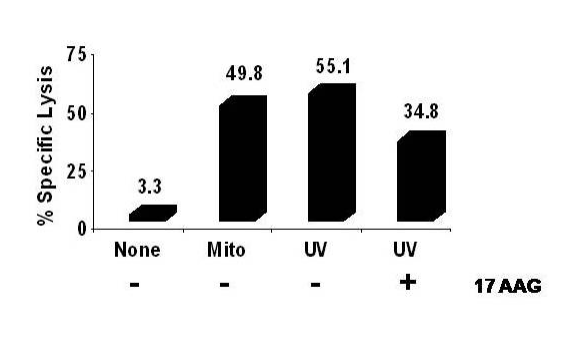
**Redirected CTL assay against RPMI following mixed culture conditions following addition of 17-AAG**. Normal donor PBMCs were primed using UV-irradiated (120 mJ/cm^2^) or mitomycin-treated (100 μg/mL) RPMI 8226 MM cells as whole cell immunogens for 28 days in 'mixed-culture conditions'. Non-primed, mito-primed, or UV-primed and PBMCs (effector cells) were then co-cultured with viable ^3^H-TdR-labeled RPMI 8226 MM cells (target cells) in a re-directed CTL assay. 17-AAG was added at 10 uM to the UV-irradiated RPMI cells for four hours and then washed away before the cells were added to the CTL culture. A standard ^3^H-TdR cytotoxicity assays was performed at E:T ratio of 50:1. Experiments were performed triplicate and expressed as the mean ± 2SEM.

In order to link the upregulation of surface expressed immunogenic HSPs to increased HSP-dependent immune recognition by CTL in the 'mixed culture' conditions, the geldanamycin derivative, 17-AAG was used. Geldanamycin and its analogue 17-allyamino, 17-demethoxygeldanamycin (17-AAG) are able to compete with ATP at the nucleotide binding site in the NH_2_-terminal domain of HSP90 and thus inhibit the peptide binding properties of HSP90 including Grp94 and does so without affecting its own expression [38,39]. As can be seen in Figure 8, 17-AAG resulted in a 37% reduction in CTL recognition of UV-primed RPMI. Although not complete, this suggests that a portion of the recognition by CTL of UV primed RPMI cells is a result of HSP-90's client protein properties.

## Conclusion

Successful cancer immunotherapy requires the reconstitution of host tumor immunosurveillance, and the production of durable cures through induction of immunological memory [40]. Although both humoral and cellular vaccines have been shown to produce cures in the short term, durable long-term cures are only possible with cell-based vaccines (e.g. CTL vaccines), because immunological memory is a cellular function. Unfortunately, the use of autologous CTLs as cellular anti-tumor vaccines for the treatment of MM has been associated with a lack of clinical efficacy; and this is widely thought to be due to the deletion of critical CTL clones that specifically target the tumor [35,41,42]. Although this may theoretically be overcome by the use of allogeneic CTL anti-tumor vaccines, this form of immunotherapy is frequently associated with limited [43] to significant [44] graft-versus-host-disease (GVHD), and may pose real dangers to the patient.

A special form of anti-cancer vaccination strategy involves the introduction of non-cellular tumor rejection molecules or Ag into a mammalian host to induce cellular immune responses. This vaccination strategy has previously been shown to be effective in promoting tumor recognition and rejection in both autologous as well as allogeneic mice [18,19]. The principal molecules mediating this process are the HSP molecular chaperones, especially TRA-1. Tumor rejection Ag-1 is a HSP [18], which has been shown to mediate tumor rejection in various cancers [19]. These molecules act to chaperone tumor-specific peptides to sites in the body where tumor Ag is most effectively presented, thereby inducing tumor recognition and tumor-specific CTL (both CD8^+ ^as well as NK cells) activation. Ultraviolet irradiation has also been shown to result in the presentation of cellular antigens. In fact, high doses of UV-B irradiation induces proinflammatory apoptosis and necrosis, where the production of inflammatory cytokines is accompanied by exposure and release of autoantigens and autoimmune disease [45].

In the present study, UV-irradiation was used to enhance the immunogenicity of MM cells while at the same time inducing apoptosis of the tumor cells. Ultraviolet light-induced Fas expression may serve to target stress-injured cells for removal by FasL-bearing cells or by FasL produced by the cells themselves in response to the stimuli, and may represent a general function of the Fas/FasL pathway in facilitating the apoptosis and elimination of undesirable or harmful cells [46]. However, UV-irradiation also appears to engage the apoptotic axis of TNFR1 [47], and appears to involve initial formation of the Fas-FADD-caspase-8 death complex in an FasL-independent manner [48]. Some types of chemotherapeutic drugs such as anthracyclins, as well as UV-C irradiation, can lead to the cell surface expression of calreticulin (CRT) which has recently been shown to confer anti-tumor immunogenicity to otherwise less immunogenic tumor cells [32]. It will be interesting to see if UV- irradiation of MM cells, as was done in this study, also leads to the surface expression of CRT. Furthermore, the expression of costimulatory molecules on dendritic cells (DC) is upregulated after co-incubation with UV-irradiated tumor cells, and UV-irradiated tumor cells-pulsed DCs stimulated allogeneic T lymphocytes more efficiently than DCs pulsed with γ-irradiated cells [49]. This increase in the ability of human cancer cells to induce CTLs by UV-irradiation has been found to be independent of the corresponding effect on histocompatibility locus Ag (HLA) expression [50]. In parallel with the induction of tumor cell immunogenicity, UV-irradiation made tumor cells more sensitive to natural killer cell-mediated cytotoxicity and to lysis by TNF, suggesting that immunogenicity and TNF sensitivity are two independent UV-induced properties [51]. It is important to note however, that the effects of mitomycin C treatment on MM cells did not alter the Grp94 or Grp78 expression significantly nor did it result in early induction of apoptosis (data not shown) and thus it served as our control in our CTL assays. The effects of 17-AAG were not tested on mitomycin treated MM cells.

When allogeneic PBMCs were exposed to these UV-irradiated MM cells, through the process of repetitive priming, CD8^+ ^CTLs were induced. Furthermore, these CTLs demonstrated strong tumor-specific activity. These data therefore confirm that not only were cytolytic NK cells preferentially induced, but that these CTLs were functionally reactive in targeting co-cultured tumor cells as well. In addition, since CTLs are widely thought to be the principal mediators of host tumor immunosurveillance [7], these data further suggest that this vaccination strategy could both reconstitute host tumor immunosurveillance as well as mediate immunological memory; i.e. be the 'Holy Grail' of cancer immunotherapy.

In conclusion, our study has shown in a functional way that it is feasible to devise an autologous cell-based anti-cancer vaccine using UV-irradiated MM cells as immunogens. Primed CTLs were associated with significant eradication of co-cultured MM cells, demonstrating in a functional and efficacious method to promote tumor rejection. Finally, since UV-irradiation increases HSP expression, we therefore postulate that the mechanism for CTL priming involves the induction of tumor peptide-loaded HSP expression on the surface of UV-irradiated MM cells, which in turn preferentially induced cytolytic T cell activation. Our study, therefore, provides the framework for the development of preclinical and clinical studies using anti-tumor cytolytic T cell vaccines for treating MM, and in which UV-irradiated tumor cells are used as immunogens.

## List of abbreviations

CTL: cytolytic T lymphocytes; E to T: effector to target; HSPs: Heat Shock Proteins; mAbs: monoclonal antibodies; MM: multiple myeloma; TAA: tumor associated antigen; TRA-1: tumor rejection antigen-1; UV: ultraviolet irradiation; 17 AAG: 17-allylamide-17-demethoxygeldanamycin.

## Authors' contributions

Both CAG and WYKH contributed equally to this work. CKP, GC and MA carried out the biochemical assays as well as some of the immunoassays. CAG and WYKH participated in the design of the study and performed the statistical analysis. CAG, WYKH and GT conceived of the study, and participated in its design and coordination and helped to draft the manuscript. All authors read and approved the final manuscript.
